# Global Renal Gene Expression Profiling Analysis in B_2_-Kinin Receptor Null Mice: Impact of Diabetes

**DOI:** 10.1371/journal.pone.0044714

**Published:** 2012-09-18

**Authors:** Miran A. Jaffa, Firas Kobeissy, Moustafa Al Hariri, Hussein Chalhoub, Assaad Eid, Fuad N. Ziyadeh, Ayad A. Jaffa

**Affiliations:** 1 Epidemiology and Population Health Department, Faculty of Health Sciences, American University of Beirut, Beirut, Lebanon; 2 Departments of Biochemistry and Molecular Genetics, American University of Beirut, Beirut, Lebanon; 3 Anatomy, Cell Biology and Physiological Sciences, Faculty of Medicine, American University of Beirut, Beirut, Lebanon; 4 Department of Medicine, Medical University of South Carolina, Charleston, South Carolina, United States of America; Bristol Heart Institute, University of Bristol, United Kingdom

## Abstract

Diabetic nephropathy (DN), the leading cause of end-stage renal failure, is clinically manifested by albuminuria and a progressive decline in glomerular filtration rate. The risk factors and mechanisms that contribute to the development and progression of DN are still incompletely defined. To address the involvement of bradykinin B_2_-receptors (B_2_R) in DN, we used a genome wide approach to study the effects of diabetes on differential renal gene expression profile in wild type and B_2_R knockout (B_2_R^−/−^) mice. Diabetes was induced with streptozotocin and plasma glucose levels and albumin excretion rate (AER) were measured at predetermined times throughout the 23 week study period. Longitudinal analysis of AER indicated that diabetic B_2_R^−/−^D null mice had a significantly decreased AER levels compared to wild type B_2_R^+/+^D mice (P = 0.0005). Results from the global microarray study comparing gene expression profiles among four groups of mice respectively: (B_2_R^+/+^C, B_2_R^+/+^D, B_2_R^−/−^C and B_2_R^−/−^D) highlighted the role of several altered pathological pathways in response to disruption of B_2_R and to the diabetic state that included: endothelial injury, oxidative stress, insulin and lipid metabolism and inflammatory process with a marked alteration in the pro-apoptotic genes. The findings of the present study provide a global genomics view of biomarkers that highlight the mechanisms and putative pathways involved in DN.

## Introduction

Diabetic nephropathy (DN) is a major health epidemic and is the main cause of morbidity and mortality in diabetes. It is the single most common cause of end-stage renal failure [1], [2]. A very characteristic and initial event of the development of DN is glomerulosclerosis, which is featured by increased thickness of the glomerular basement membrane, and widening of the mesangium with accumulation of extracellular matrix (ECM). Furthermore, the degree of mesangial expansion is strongly related to the clinical manifestations of diabetic nephropathy, such as albuminuria and decreased glomerular filtration rate [3], [4]. Even though inherent susceptibility seems to influence the rate at which glomerular injury develops, hyperglycemia seems to be the primary driving force for cellular damage [5]. In this regard, intensive control of glycemia in type I diabetic patients was associated with a significant reduction in the development and progression of nephropathy [6].

Although, the underlying biochemical and cellular mechanisms that promote renal injury in diabetes are still undefined, accumulating evidence supports a relationship between the activity of the kallikrein-kinin system (KKS) and renal impairment. It has been shown that type I diabetic patients with hyperfiltration as well as diabetic rats with increased glomerular filtration rate (GFR) and renal plasma flow (RPF) are associated with increased active kallikrein excretion rate [7], [8]. In addition, treatment of hyperfiltering diabetic rats with aprotinin, a kallikrein inhibitor, or with a B_2_-kinin receptor (B_2_R) antagonist, increases the renal vascular resistance and reduces GFR and RPF [9]. Furthermore, previous findings from our lab have shown that increased plasma prekallikrein activity is associated with increased albumin excretion rate; these data have been demonstrated in DCCT/EDIC-cohort of type 1 diabetic patients [10].

While most of the physiological actions of the KKS are attributed to the generation of BK and activation of B_2_R, the intracellular signaling pathways initiated upon activation of B_2_R leading to expression of prosclerotic factors that ultimately result in glomerular injury are just beginning to be defined. Activation of B_2_R by BK results in marked induction of connective tissue growth factor (CTGF), collagen I and transforming growth factor-β type II receptor (TGF-ßRII) in mesangial cells. Inhibition of B_2_R by Icatibant significantly reduced the increase in collagen I and CTGF mRNA levels in response to BK challenge [11]. Of interest, it has been shown that the glomerular expression of B_2_Rs are increased in diabetes and a targeted deletion B_2_R protects against the development of DN [12], [13]. Furthermore, diabetic B_2_R^−/−^ null mice display reduced albumin excretion rate (AER), as well as reduced glomerular and tubular injury compared to diabetic B_2_R^+/+^ mice [13].

In this study, we employed a global microarray analysis coupled with systems biology study to investigate the differential gene expression in wild type control (B_2_R^+/+^) and diabetic (B_2_R^+/+^D) mice as well as in B_2_R knockout-control (B_2_R^−/−^) mice and in B_2_R knockout-diabetic (B_2_R^−/−^D) mice in order to identify candidate genes that may be involved in the development of diabetic nephropathy. The objective of our study was to determine 1) whether deletion of B_2-_receptors will result in alteration in specific gene expression profiles whose specific functions can shed light on the role(s) of B_2-_receptors, and 2) whether diabetes will result in differences in the patterns of gene expression and pathways between B_2_R^+/+^D and B_2_R^−/−^D mice that can be linked to the pathological manifestation observed after the induction of DN.

## Methods

### Study Design

To address the contribution of B_2_R to the development of diabetic nephropathy, we studied B_2_R knockout mice (B_2_R^−/−^) and their wild type littermates (B_2_R^+/+^). Male B_2_R^−/−^ mice (strain # B6 129S-BdKrb2, Jackson Laboratories, Bar Harbor, ME) and B_2_R^+/+^ mice (strain # B6 129 SF2/J, Jackson Laboratories, Bar Harbor, ME) weighing 20–30 g were used in our studies. Mice were housed three per cage in a light and temperature controlled room and had free access to food and water. Diabetes was induced by daily intraperitoneal injection of streptozotocin (50mg/kg body weight) for 3–5 days. Diabetes was confirmed in STZ-treated mice by tail vein plasma glucose levels. We used a total of 12 mice for this study divided into 4 groups, 3 mice in each group. Group 1, wild type non-diabetic-controls (B_2_R^+/+^C); group 2, wild type-diabetic (B_2_R^+/+^ D); group 3, B_2_R knockout-control (B_2_R^−/−^C) and group 4, B_2_R knockout-diabetic (B_2_R^−/−^D). Glucose levels and body weights were measured at predetermined intervals to characterize the diabetic state and to ensure adequate metabolic control. Every week mice were placed in metabolic cages (Nalgene) for 24 h to acclimate, and then 24h urine collections were obtained from all mice to measure albumin excretion rate. The mice were sacrificed 6 months after the induction of diabetes. The studies were done in line with the Guide for the Care and Use of laboratory Animals published by the National Institutes of Health (NIH Publication No 85–23, revised 1996). The study was approved by the Institutional Animal Care and Use Committee at the Medical University of South Carolina.

**Figure 1:**
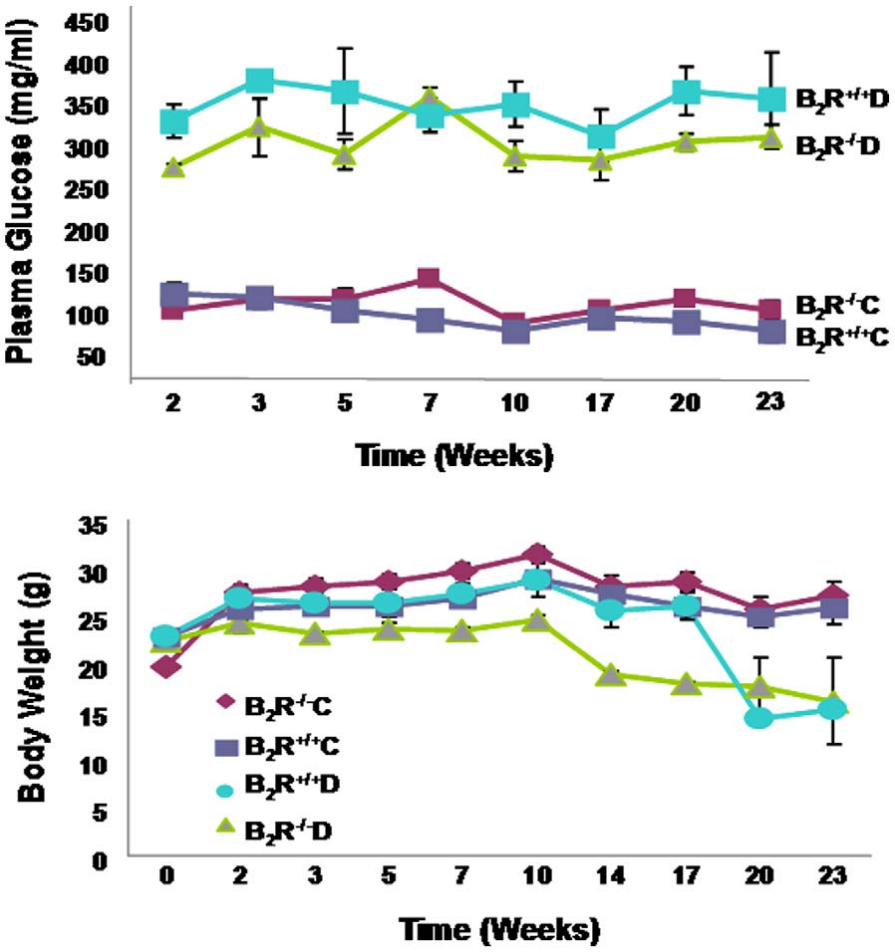
Plasma glucose levels (A) and body weights (B) in diabetic (B_2_R^+/+^D and B_2_R^−/−^D) and control (B_2_R^+/+^C and B_2_R^−/−^C) mice. (**A**) Plasma glucose levels were significantly increased two weeks after STZ injection in both diabetic groups (B_2_R^+/+^D and B_2_R^−/−^D) compared to B_2_R^+/+^C and B_2_R^−/−^C (P<0.001) and remained significantly elevated for the duration of the study. (**B**) Initial body weights were not significantly different between diabetic and control mice. However, B_2_R^−/−^D mice had significantly reduced bodyweight after 14 weeks and B_2_R^+/+^D after 20 weeks compared with B_2_R^+/+^ C and B_2_R^−/−^ C mice and this reduction in body weight was maintained for the duration of the study (P<0.001 vs. B_2_R^+/+^C and B_2_R^−/−^C).

### RNA Extraction

Kidneys from control and diabetic mice (B_2_R^+/+^C, B_2_R^+/+^D, B_2_R^−/−^C and B_2_R^−/−^D mice, n = 3 per group) were removed under anesthesia and cortexes were cut off to extract RNA. For RNA extraction and purification, a method combined Trizol (Cat. No.15596-018, Invitrogen Life Technologies) and RNeasy Midi Kit (Cat. No.75144, QIAGEN) for total RNA isolation from animal tissue was used. Briefly, the cortexes were homogenized using an appropriate volume of Trizol (1ml of Trizol/100 mg tissue). Then chloroform (0.2 ml/1 ml Trizol used) was added to separate the aqueous phase from protein phase. Total RNA was dissolved in the aqueous phase. RNA purification followed the protocol of RNeasy kit handbook. The RNA concentration was determined in a spectrophotometer (ultraspec III, Pharmacia) by absorbance at 260 nm. The ratio of A260 to A280 was calculated to check the purification of RNA, and the rRNA ratio of 28S/18S using 2100 Bioanalyzer (Aglilent) was measured to check the quality of RNA.

**Figure 2:**
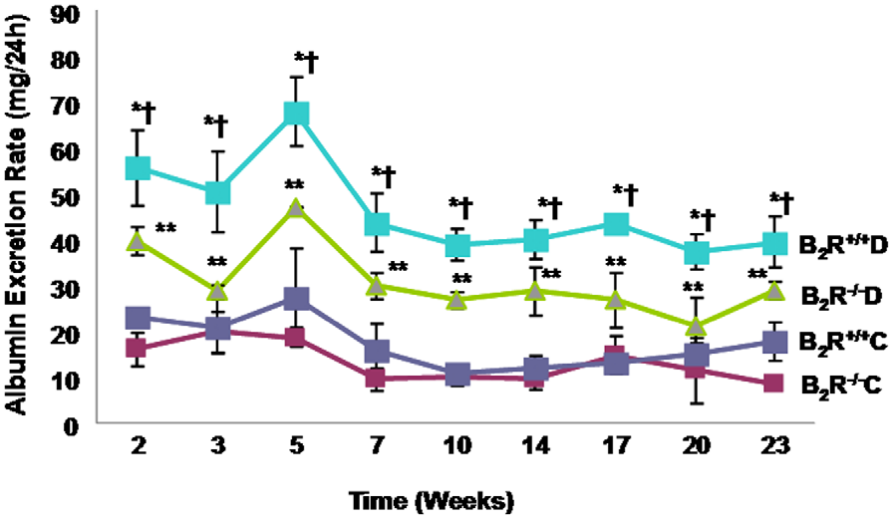
Albumin excretion rate (AER) in diabetic (B_2_R^+/+^D and B_2_R^−/−^D) and control (B_2_R^+/+^C and B_2_R^−/−^C) mice. AER was significantly higher in B_2_R^+/+^D mice compared to B_2_R^−/−^ D (†P<0.05) and to B_2_R^+/+^C and or B_2_R^−/−^C (*P<0.001), as early as two weeks after induction of diabetes and remained elevated for the duration of the study period.

### Synthesis of Double-stranded cDNA from Total RNA

Total RNA (10 µg) from each sample was used to synthesize ds-cDNA. In primer hybridization, 10 µg of RNA, T7-(dT)_24_ primer (100 pmol/ul, HPLC purified) and DEPC-H_2_O were added to the tube and incubated at 70°C for 10 min. Next, 5× first strand cDNA buffer 4 µl, DTT (0.1 M) 2 µl dNTP (10 mM) were added to each tube, incubated at 42°C for 2 min. and followed by addition of SuperScrip II RT (200 U/µl) 2 µl and incubated at 42°C for 1 hour to synthesize the first strand of cDNA. The final volume for the first strand cDNA synthesis was 20 µl. In order to synthesis the second strand, the following reagents were added to the first strand synthesis tube: DEPC-treated water 91 µl, 5× second strand cDNA reaction buffer 30 µl, 10 mM dNTP mix 3 µl, 10 U/µl *E*.*coli* DNA ligase, 10 U/µl *E*.*coli* DNA polymerase I 4 µl and 2 U/µl *E*.*coli* RNase H. The final volume of the second strand reaction was 150 µl. The reaction tubes were incubated at 16°C for 2 hours in a cooling water bath. After the incubation, 2 µl of [10 U] T4 DNA polymerase was added to the reaction tube, incubated at 16°C for 5 min, followed by addition of 10 µl of 0.5 M EDTA to complete synthesis of the second cDNA strand.

**Figure 3:**
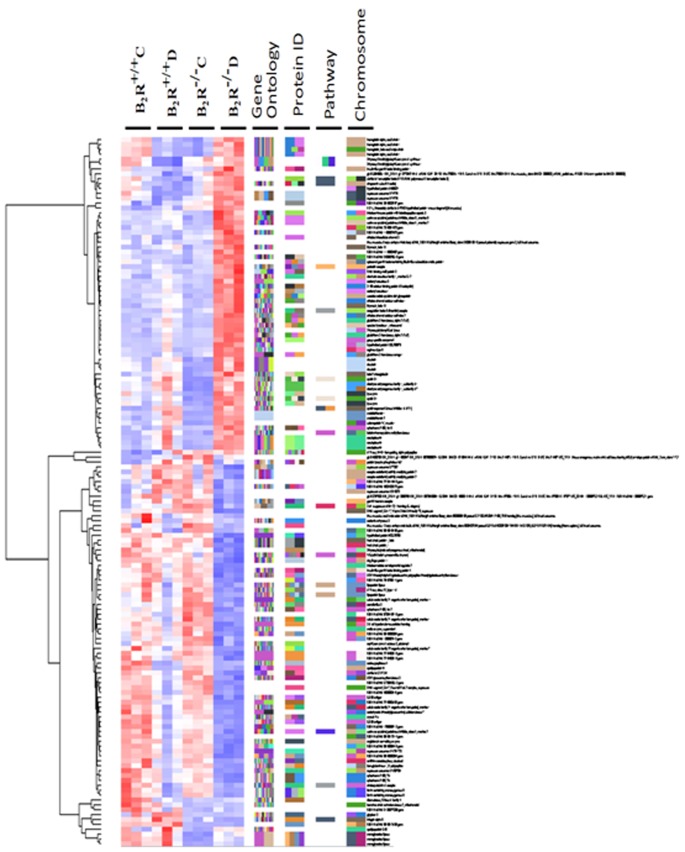
Hierarchical clustering of gene expression in the kidney among four groups of mice: B_2_R^+/+^C, B_2_R^+/+^D, B_2_R^−/−^C and B_2_R^−/−^D. Each column represents one sample, and the color bars represent the median value of three array experiments for an individual mouse for that gene.

### Synthesis of Biotin-labeled cRNA

Before Synthesis of biotin-labeled cRNA, double-strand cDNA was cleaned according to the GeneChip Sample Cleanup Module. The following reagents were used in the final reaction volume (40 µl): 4 µl of 10×HY reaction buffer, 4 µl of 10×Biotin-labeled ribonucleotides, 4 µl of 10×DTT, 4 µl 10×RNase inhibitor mix, 2 µl 20×T7 RNA polymerase and distilled water. All of the reagents were mixed and incubated at 37°C for 5 hours, with gentle mixing of the tube every 30 min. The biotin-labeled cRNA was cleaned according to the GeneChip Sample Cleanup Module before quantification.

**Figure 4:**
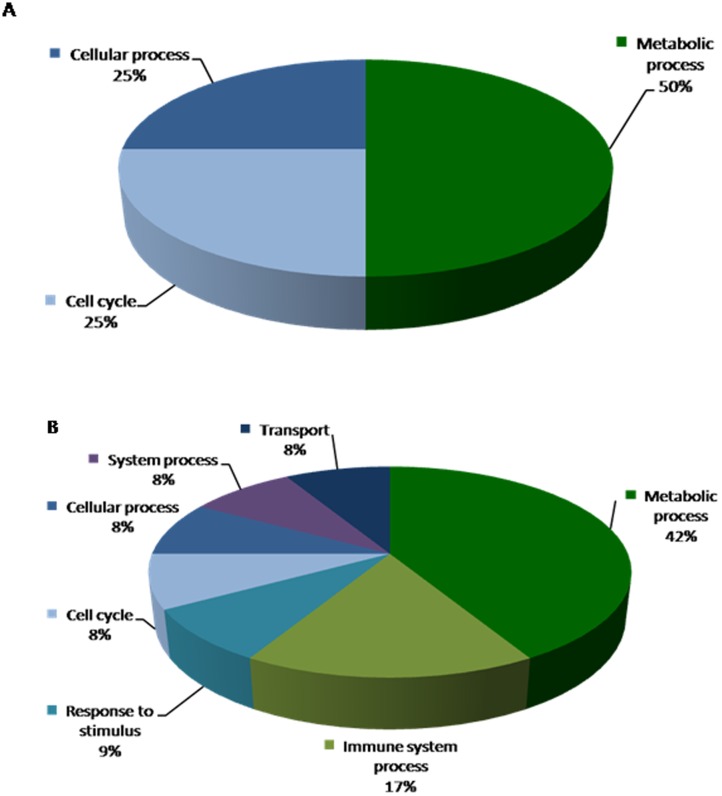
Biological processes depicting genes that are altered in response to B_2_R disruption are shown in pie chart. Data compares genes altered in B_2_R^−/−^C vs. B_2_R^+/+^C (**A**) upregulated genes and (**B**) downregulated genes.

**Table 1:** Upregulated and Downregulated Genes in B_2_R^−/−^C vs. B_2_R^+/+^C.

Accession ID	Gene	Gene ID	Fold Change	P value
NM_018731	Atp4a ATPase, H+/K+ exchanging, gastric, alpha polypeptide	11944	2.77	0.001678
AK007618	Ak3 adenylate kinase 3	56248	1.85	0.017419
NM_001164745	Ptp4a2 protein tyrosine phosphatase 4a2	19244	2.97	0.028458
NM_012032	Serinc3 serine incorporator 3	26943	2.14	0.013514
NM_013467	Aldh1a1 aldehyde dehydrogenase family 1, subfamily A1	11668	−4.13	0.003764
BC027434	Hbb-b2 hemoglobin, beta adult minor chain	15130	−2.42	0.002817
NM_008218	Hba-a1 hemoglobin alpha, adult chain 1	15122	−2.54	0.00193
NM_011921	Aldh1a7 aldehyde dehydrogenase family 1, subfamily A7	26358	−2.35	0.005015
BC005569	Rnase4 ribonuclease, RNase A family 4	58809	−4.28	0.00286
AF031467	Bcat2 branched chain aminotransferase 2, mitochondrial	12036	−2.49	0.047991
NM_011844	Mgll monoglyceride lipase	23945	−3.01	0.028657
BC027279	Blvrb biliverdin reductase B (flavin reductase (NADPH))	233016	−1.94	0.016502
NM_001003953	Kdm2b lysine (K)-specific demethylase 2B	30841	−1.81	0.001347

### cRNA Fragmentation and Microarray Procedure

To reach a final concentration of 1 µg/µl, 20 µg cRNA and 8 µl of 5×fragmentation buffer were incubated at 94°C for 35 min. A total of 15 µl of each sample (1.0 µg/µl) was used for preparation of hybridization cocktail that was loaded onto the GeneChips (Mouse Expression Array 430 A, Affymetrix) and hybridized for 16 h at 45°C in the Affymetrix GeneChip hybridization oven 640. Following this, the chips were loaded into the Affymetrix GeneChip Fluidics Station 400 with double stain antibody amplification solution for washing and staining. Finally, the GeneChips were scanned using the Hewlett Packard GeneArray Scanner 2500.

Expression values were derived using RMA (for normalization and background subtraction) as executed by the software RMAexpress (University of California, Berkeley). Expressed genes were determined according to the following criterion: any gene for which a sample had an average detection p-value (MASS) >0.04 (standard threshold for MASS “presence” call); all other genes were excluded from further consideration. RMA expression values were converted from log-base 2 and imported into dchip. Dchip was used to perform comparisons for all desired group comparisons. Criteria for comparison were: Fold change of 1.8; 90% confidence bound of fold change was used; T-test with p-value <0.05; false discovery rate was calculated as the median number genes discovered in 50 iterations of permutated samples.

**Figure 5:**
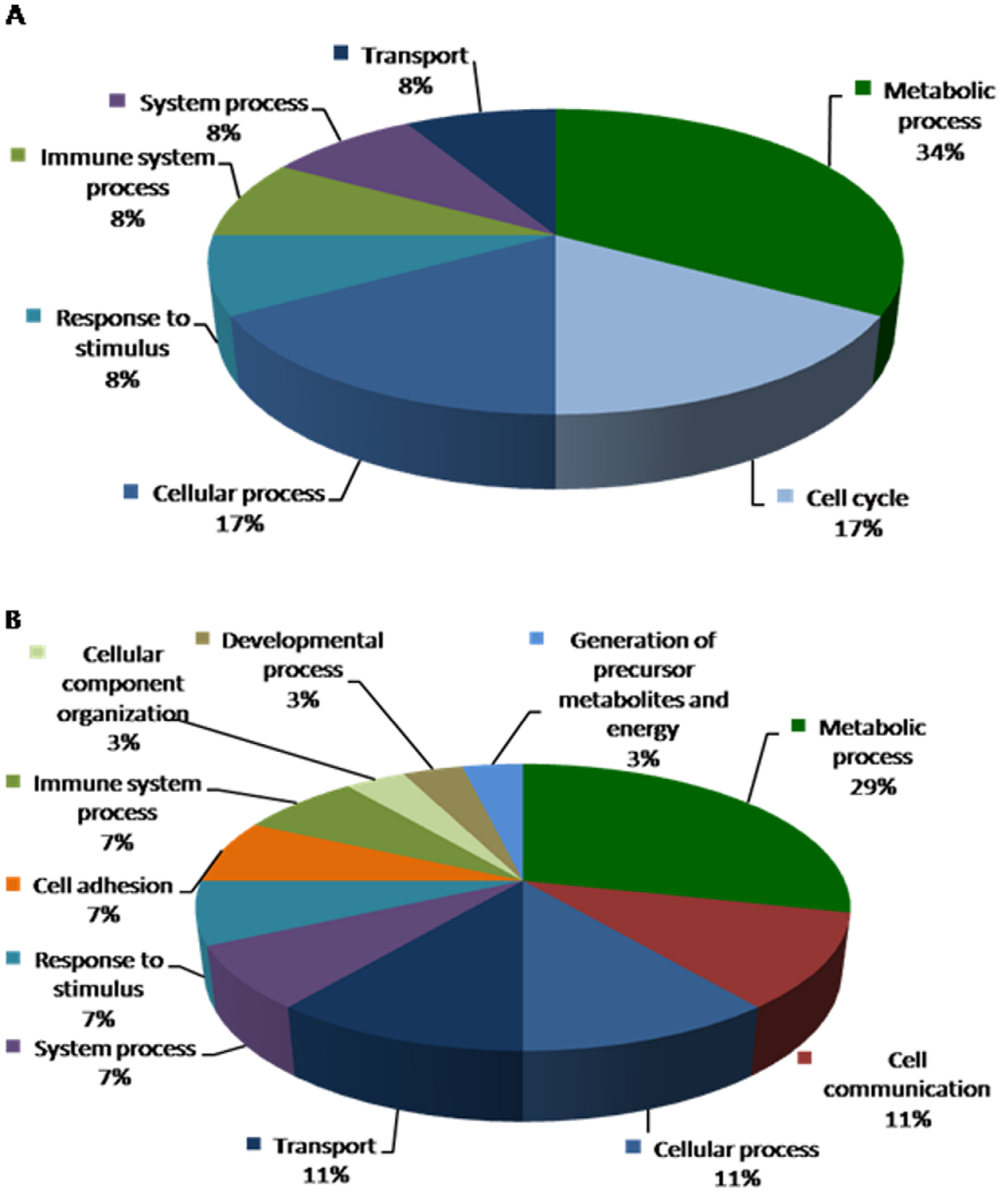
Biological processes depicting genes that are altered in response to diabetes in wild type control mice are shown in pie chart. Data compares genes altered in B_2_R^+/+^D vs. B_2_R^+/+^C (**A**) upregulated genes and (**B**) downregulated genes.

**Table 2:** Upregulated and Downregulated Genes in B_2_R^+/+^D vs. B_2_R^+/+^C.

Accession ID	Gene	Gene ID	Fold Change	P value
NM_019659	Kcnj1 potassium inwardly-rectifying channel, subfamily J, member 1	56379	2.1	0.005281
NM_011819	Gdf15 growth differentiation factor 15	23886	1.82	0.031049
AK007630	Cdkn1a cyclin-dependent kinase inhibitor 1A (P21)	12575	4.61	0.000166
AK008108	Sulf2 sulfatase 2	72043	1.91	0.027765
AK013376	Aplp2 amyloid beta (A4) precursor-like protein 2	11804	1.88	0.022803
AK007618	Ak3 adenylate kinase 3	56248	1.99	0.010866
NM_030558	Car15 carbonic anhydrase 15	80733	1.99	0.013746
AAC42082	Ccng1 cyclin G1	12450	1.91	0.007673
NM_012032	Serinc3 serine incorporator 3	26943	2.02	0.018166
BC010197	Cpe carboxypeptidase E	12876	−2.91	0.001076
NM_008321	Id3 inhibitor of DNA binding 3	15903	−1.97	0.007472
NM_013475	Apoh apolipoprotein H	11818	−2.55	0.005274
BC027434	Hbb-b2 hemoglobin, beta adult minor chain	15130	−2.17	0.003365
NM_008218	Hba-a1 hemoglobin alpha, adult chain 1	15122	−2.1	0.001414
D89669	Cyp24a1 cytochrome P450, family 24, subfamily a, polypeptide 1	13081	−2.34	0.038907
BC020534	Cckar cholecystokinin A receptor	12425	−2.28	0.032112
NM_007812	Cyp2a5 cytochrome P450, family 2, subfamily a, polypeptide 5	13087	−1.85	0.038301
BC005569	Rnase4 ribonuclease, RNase A family 4	58809	−3.31	0.002108
NM_030888	C1qtnf3 C1q and tumor necrosis factor related protein 3	81799	−2.24	0.025602
BC013343	Hpd 4-hydroxyphenylpyruvic acid dioxygenase	15445	−1.98	0.027661
S64539	Odc1 ornithine decarboxylase, structural 1	18263	−1.86	0.045764
AK011116	Hba-a1 hemoglobin alpha, adult chain 1	15122	−1.98	0.004592
AAH23851	Hmgcs1 3-hydroxy-3-methylglutaryl-Coenzyme A synthase 1	208715	−2.71	0.013067
NM_001004148	Slc13a5 solute carrier family 13 (sodium-dependent citrate transporter), member 5	237831	−1.84	0.00636
EDL41048	Id4 inhibitor of DNA binding 4	15904	−1.95	0.031173

### Real-Time PCR

Total RNA (2 µg) was converted to cDNA using MLV Reverse Transcriptase (Promega, Madison, WI) according to the manufacturer’s protocol at 37°C for 1 hr. To determine the validity of primers and appropriate Tm for Real Time PCR, the primers were first amplified in a PCR reaction to ensure that only one band is amplified. The following primers were designed so that all of the PCR products are within 75–150 bp (Integrated DNA Technologies Inc). β-actin: 5′-actgccgctcctcttcctc-3′; 5′-ccgctcgttgccaatagtga-3′; Growth hormone receptor: 5′- ttctgggaagcctcgattcaccaa-3′, 5′:cagcttgtcgttggctttcccttt-3′; Insulin growth factor binding protein-1(IGFBP1) 5′: agatcgccgacctcaagaaatgga-3′, 5′-tgttgggctgcagctaatctctct-3′; IGFBP4: 5′-tcggaaatcgaagccatccaggaa-3′, 5′-tgaagctgttgttgggatgttcgc-3′; Extracellular superoxide distmutase (EC-SOD) 5′-tgcatgcaatctgcagggtacaac-3′, 5′-aagagaaccaagccggtgatctgt-3′; Flavin containing monooxygenase 2 (FMO2) 5′-caacgcactgtctttgacgctgtt-3′, 5′-atggaaatactggcttcggaacct-3′; Glutathione-S-transferase a-2 (GSTa-2) 5′-atgacaaggactaccttgtgggca-3′, 5′-ggctggcatcaagctcttcaacat-3′. For each target gene, a standard curve was established. This was achieved by performing a series of 3-fold dilutions of the gene of interest. Negative control was made using the same volume of Rnase-free water instead of sample. The master mix was prepared as follows: 2× SYBR Green Supermix (cat. No. 170–8880, BIO-RAD) 12.5 µl, forward and reverse primer 0.25 µl respectively and ddH2O 12 µl. For each well, 22 µl of master mix was loaded first, followed by 3 µl of sample, mixed well to get total reaction volume of 25 µl. For plate setup, SYBR-490 was chosen as fluorophore. The plate was covered with a sheet of optical sealing film. PCR conditions were 95°C for 3 min, followed by 40 cycles of 95°C for 10 sec, 58°C for 1 min for ß-actin and for all the other genes 60°C for 1 min, then 95°C for 1 min, 55°C for 1 min and 100 cycles of 55°C for 10 sec. All of the reactions were done in duplicate. The correlation coefficient is between 0.98-1, PCR efficiency is between 75–130%. The mRNA levels were expressed relative to ß-actin mRNA. Realtime PCR using iCycle™ iQ optical system software (version 3.0a) was used in our studies.

**Figure 6:**
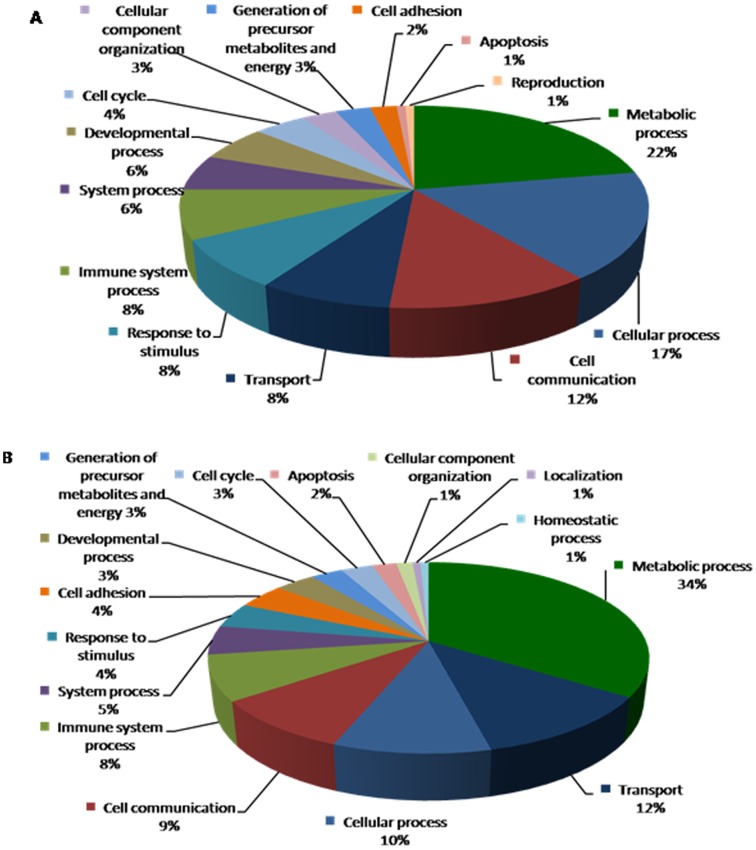
Biological processes depicting genes that are altered in response to diabetes in B_2_R^−/−^ null mice are shown in pie chart. Data compares genes altered in B_2_R^−/−^D vs. B_2_R^−/−^C (**A**) upregulated genes and (**B**) downregulated genes.

**Table 3:** Upregulated and Downregulated Genes in B_2_R^−/−^D vs. B_2_R^−/−^C.

Accession ID	Gene	Gene ID	Fold Change	P value
BC009155	Mgst1 microsomal glutathione S-transferase 1	56615	1.97	0.035109
NM_019703	Pfkp phosphofructokinase, platelet	56421	1.99	0.045538
EDL25631	Mpzl2 myelin protein zero-like 2	14012	1.86	0.035095
NM_019423	Elovl2 elongation of very long chain fatty acids (FEN1/Elo2, SUR4/Elo3, yeast)-like 2	54326	2.03	0.003224
NM_013467	Aldh1a1 aldehyde dehydrogenase family 1, subfamily A1	11668	8.21	0.015004
NM_009994	Cyp1b1 cytochrome P450, family 1, subfamily b, polypeptide 1	13078	1.84	0.022511
NM_009255	Serpine2 serine (or cysteine) peptidase inhibitor, clade E, member 2	20720	2.25	0.002624
NM_021897	Trp53inp1 transformation related protein 53 inducible nuclear protein 1	60599	1.84	0.024129
BC027434	Hbb-b2 hemoglobin, beta adult minor chain	15130	2.69	0.013965
NM_025284	Tmsb10 thymosin, beta 10	19240	1.9	0.024637
NM_007631	Ccnd1 cyclin D1	12443	2.95	0.000665
NM_007752	Cp ceruloplasmin	12870	2.49	0.007697
NM_008218	Hba-a1 hemoglobin alpha, adult chain 1	15122	3.2	0.019897
AF047838	Clca1 chloride channel calcium activated 1	12722	3.37	0.011734
BC021776	Apoc3 apolipoprotein C-III	11814	2.01	0.000641
NM_009244	Serpina1b serine (or cysteine) preptidase inhibitor, clade A, member 1B	20701	2.24	0.006778
NM_008332	Ifit2 interferon-induced protein with tetratricopeptide repeats 2	15958	2.05	0.022893
NM_001198560	H2-Q7 histocompatibility 2, Q region locus 7	15018	1.84	0.013281
NM_011921	Aldh1a7 aldehyde dehydrogenase family 1, subfamily A7	26358	3.96	0.030189
NM_013492	Clu clusterin	12759	1.92	0.000802
NM_009705	Arg2 arginase type II	11847	1.98	0.00093
D89669	Cyp24a1 cytochrome P450, family 24, subfamily a, polypeptide 1	13081	3.09	0.079614
NM_008341	Igfbp1 insulin-like growth factor binding protein 1	16006	2.81	0.042115
NM_031161	Cck cholecystokinin	12424	2.17	0.002063
NM_010281	Ggh gamma-glutamyl hydrolase	14590	2	0.012756
NM_019738	Nupr1 nuclear protein 1	56312	1.85	0.001812
NM_008935	Prom1 prominin 1	19126	2.21	0.014173
NM_031185	Akap12 A kinase (PRKA) anchor protein (gravin) 12	83397	1.86	0.00053
NM_009831	Ccng1 cyclin G1	12450	2.15	0.007653
NM_008182	Gsta2 glutathione S-transferase, alpha 2 (Yc2)	14858	2.65	0.000003
NM_011313	S100a6 S100 calcium binding protein A6 (calcyclin)	20200	3.53	0.000785
NM_011169	Prlr prolactin receptor	19116	3.04	0.026061
NM_010145	Ephx1 epoxide hydrolase 1, microsomal	13849	5.62	0.000013
NM_013602	Mt1 metallothionein 1	17748	2.96	0.003938
NM_009256	Serpinb9 serine (or cysteine) peptidase inhibitor, clade B, member 9	20723	1.88	0.031251
NM_001166409	Rbm3 RNA binding motif protein 3	19652	2.57	0.034705
NM_009162	Scg5 secretogranin V	20394	2.08	0.031656
NM_013590	Lyz1 lysozyme 1	17110	2.11	0.004781
BC010291	Ifitm3 interferon induced transmembrane protein 3	66141	2.1	0.001773
AK007630	Cdkn1a cyclin-dependent kinase inhibitor 1A (P21)	12575	4.38	0.018945
BC010747	Cyp4a10 cytochrome P450, family 4, subfamily a, polypeptide 10	13117	2.97	0.008145
BC019601	Wsb1 WD repeat and SOCS box-containing 1	78889	2.08	0.017203
AK011116	Hba-a1 hemoglobin alpha, adult chain 1	15122	2.87	0.004126
NM_008630	Mt2 metallothionein 2	17750	2.3	0.000286
AK002562	Reep6 receptor accessory protein 6	70335	1.83	0.006197
AK019319	Apoe apolipoprotein E	11816	2.03	0.013394
NM_009964	Cryab crystallin, alpha B	12955	1.98	0.008461
NM_009700	Aqp4 aquaporin 4	11829	2.24	0.04758
NM_011403	Slc4a1 solute carrier family 4 (anion exchanger), member 1	20533	1.88	0.005501
NM_028071	Cotl1 coactosin-like 1 (Dictyostelium)	72042	2.52	0.045007
NM_033521	Laptm4b lysosomal-associated protein transmembrane 4B	114128	2.24	0.022123
NM_017372	Lyz2 lysozyme 2	17105	3.11	0.0077
NM_019989	Sh3bgrl SH3-binding domain glutamic acid-rich protein like	56726	2.1	0.02642
NM_001206367	Gsn gelsolin	227753	1.82	0.004725
NM_001039392	Tmsb10 thymosin, beta 10	19240	2.41	0.009177
NM_010169	F2r coagulation factor II (thrombin) receptor	14062	2.66	0.008048
NM_007569	Btg1 B-cell translocation gene 1, anti-proliferative	12226	1.9	0.027652
NM_013492	Clu clusterin	12759	3.04	0.023634
NM_010664	Krt18 keratin 18	16668	2.33	0.032815
NM_009242	Sparc secreted acidic cysteine rich glycoprotein	20692	2.77	0.01031
NM_021281	Ctss cathepsin S	13040	1.94	0.003757
NM_007631	Ccnd1 cyclin D1	12443	2.36	0.01662
NM_011579	Tgtp1 T-cell specific GTPase 1	21822	2.11	0.048236
NM_010501	Ifit3 interferon-induced protein with tetratricopeptide repeats 3	15959	2.4	0.043235
NM_019975	Hacl1 2-hydroxyacyl-CoA lyase 1	56794	2.68	0.000059
NM_012006	Acot1 acyl-CoA thioesterase 1	26897	2.11	0.001113
NM_009735	B2m beta-2 microglobulin	12010	2.8	0.025118
NM_009517	Zmat3 zinc finger matrin type 3	22401	1.93	0.005952
NM_054102	Ivns1abp influenza virus NS1A binding protein	117198	1.98	0.02154
NM_009254	Serpinb6a serine (or cysteine) peptidase inhibitor, clade B, member 6a	20719	2.45	0.016523
NM_011844	Mgll monoglyceride lipase	23945	2.2	0.021782
AF177041	Akr1c12 aldo-keto reductase family 1, member C12	622402	2.06	0.010183
NM_016668	Bhmt betaine-homocysteine methyltransferase	12116	3.04	0.00654
NM_010379	H2-Ab1 histocompatibility 2, class II antigen A, beta 1	14961	1.86	0.01325
NM_010169	coagulation factor II (thrombin) receptor	14062	2.02	0.003274
AF263458	Plac8 placenta-specific 8	231507	1.84	0.001426
BC008184	Aldoc aldolase C, fructose-bisphosphate	11676	2	0.008866
BC027340	Lyplal1 lysophospholipase-like 1	226791	1.8	0.001368
BC012874	Serpina1b serine (or cysteine) preptidase inhibitor, clade A, member 1B	20701	2.34	0.024747
NM_009735	B2m beta-2 microglobulin	12010	1.88	0.015559
AK011116	Hba-a1 hemoglobin alpha, adult chain 1	15122	2.52	0.003324
NM_013492	Clu clusterin	12759	2.45	0.011136
NM_001042611	Cp ceruloplasmin	12870	4.02	0.000586
NM_010362	Gsto1 glutathione S-transferase omega 1	14873	2.53	0.001403
NM_009369	Tgfbi transforming growth factor, beta induced	21810	1.82	0.021522
NM_011701	Vim vimentin	22352	1.93	0.022829
NM_008538	Marcks myristoylated alanine rich protein kinase C substrate	17118	1.83	0.018183
NM_007620	Cbr1 carbonyl reductase 1	12408	3.36	0.016972
AF108501	Clca2 chloride channel calcium activated 2	80797	4.01	0.007099
NM_013470	Anxa3 annexin A3	11745	1.81	0.022226
NM_009156	Sepw1 selenoprotein W, muscle 1	20364	2.02	0.001731
NM_008509	Lpl lipoprotein lipase	16956	−3.3	0.000892
BC010197	Cpe carboxypeptidase E	12876	−2.11	0.000013
AF145253	Sec61a1 Sec61 alpha 1 subunit (S. cerevisiae)	53421	−2.13	0.005158
NM_007823	Cyp4b1 cytochrome P450, family 4, subfamily b, polypeptide 1	13120	−1.92	0.00051
BC013477	Adh1 alcohol dehydrogenase 1 (class I)	11522	−1.92	0.007629
NM_013560	Hspb1 heat shock protein 1	15507	−2.56	0.004221
NM_013475	Apoh apolipoprotein H	11818	−2.66	0.003569
BC021352	Plod2 procollagen lysine, 2-oxoglutarate 5-dioxygenase 2	26432	−1.98	0.012487
NM_029550	Keg1 kidney expressed gene 1	64697	−1.85	0.000586
NM_008766	Slc22a6 solute carrier family 22 (organic anion transporter), member 6	18399	−1.8	0.001602
NM_007376	Pzp pregnancy zone protein	11287	−2.24	0.022851
NM_008878	Serpinf2 serine (or cysteine) peptidase inhibitor, clade F, member 2	18816	−2.54	0.001222
NM_010007	Cyp2j5 cytochrome P450, family 2, subfamily j, polypeptide 5	13109	−2.12	0.042784
NM_011435	Sod3 superoxide dismutase 3, extracellular	20657	−2	0.023677
NM_021788	Sap30 sin3 associated polypeptide	60406	−1.82	0.005361
NM_013478	Azgp1 alpha-2-glycoprotein 1, zinc	12007	−2.14	0.004734
AW105741	Slc16a2 solute carrier family 16 (monocarboxylic acid transporters), member 2	20502	−2.32	0.007439
BC012637	Aadat aminoadipate aminotransferase	23923	−1.97	0.007973
NM_008129	Gclm glutamate-cysteine ligase, modifier subunit	14630	−2.06	0.015486
BC016885	Ugt8a UDP galactosyltransferase 8A	22239	−2.77	0.004372
L27424	Timp3 tissue inhibitor of metalloproteinase 3	21859	−2.34	0.006506
NM_027884	Tns1 tensin 1	21961	−2.12	0.049487
NM_013797	Slco1a1 solute carrier organic anion transporter family, member 1a1	28248	−11.51	0.014794
NM_008079	Galc galactosylceramidase	14420	−2.1	0.017597
NM_030721	Acox3 acyl-Coenzyme A oxidase 3, pristanoyl	80911	−2.68	0.006701
NM_007825	Cyp7b1 cytochrome P450, family 7, subfamily b, polypeptide 1	13123	−5.04	0.009947
NM_015804	Atp11a ATPase, class VI, type 11A	50770	−2.76	0.012782
BC020534	Cckar cholecystokinin A receptor	12425	−2.11	0.006061
AB008174	Hnf1b HNF1 homeobox B	21410	−1.94	0.032091
NM_008016	Mpp6 membrane protein, palmitoylated 6 (MAGUK p55 subfamily member 6)	56524	−2.17	0.00856
NM_053097	Cml3 camello-like 3	93674	−2.48	0.001103
NM_010232	Fmo5 flavin containing monooxygenase 5	14263	−2.66	0.000267
NM_008173	Nr3c1 nuclear receptor subfamily 3, group C, member 1	14815	−1.86	0.006131
NM_008261	Hnf4a hepatic nuclear factor 4, alpha	15378	−2.08	0.004817
NM_010517	Igfbp4 insulin-like growth factor binding protein 4	16010	−2.37	0.00307
NM_010496	Id2 inhibitor of DNA binding 2	15902	−1.88	0.004033
NM_009203	Slc22a12 solute carrier family 22 (organic anion/cation transporter), member 12	20521	−2.13	0.044714
AK004192	Cd36 CD36 antigen	12491	−2.07	0.000977
NM_001160404	Galnt1 UDP-N-acetyl-alpha-D-galactosamine:polypeptide N-acetylgalactosaminyltransferase 1	14423	−2.22	0.001029
NM_009467	Ugt2b5 UDP glucuronosyltransferase 2 family, polypeptide B5	22238	−1.99	0.005327
NM_010279	Gfra1 glial cell line derived neurotrophic factor family receptor alpha 1	14585	−1.86	0.029282
BC003451	Mat2a methionine adenosyltransferase II, alpha	232087	−2.03	0.000527
BC019374	Gclc glutamate-cysteine ligase, catalytic subunit	14629	−2.18	0.00284
BC025936	Cyp4a12a cytochrome P450, family 4, subfamily a, polypeptide 12a	277753	−3.57	0.000548
U68542	Cux1 cut-like homeobox 1	13047	−2.1	0.024224
BC013521	Anxa13 annexin A13	69787	−1.94	0.041203
AY038079	Fbxw11 F-box and WD-40 domain protein 11	103583	−2.24	0.009461
BC003476	Cd74 CD74 antigen (invariant polypeptide of major histocompatibility complex, class II antigen-associated)	16149	−1.9	0.012558
AB022340	Acsm3 acyl-CoA synthetase medium-chain family member 3	20216	−3.76	0.001304
AF213670	Mlx MAX-like protein X	21428	−2.08	0.007919
NM_001164099	Add3 adducin 3 (gamma)	27360	−1.84	0.002187
BC027063	Bdh1 3-hydroxybutyrate dehydrogenase, type 1	71911	−2.16	0.001797
BC023060	Efemp1 epidermal growth factor-containing fibulin-like extracellular matrix protein 1	216616	−2.16	0.001651
S64539	Odc1 ornithine decarboxylase, structural 1	18263	−2.3	0.033305
NM_009202	Slc22a1 solute carrier family 22 (organic cation transporter), member 1	20517	−1.95	0.002763
AK003232	Cbr3 carbonyl reductase 3	109857	−3.48	0.045187
AK009736	Gpr137b-ps G protein-coupled receptor 137B, pseudogene	664862	−2.01	0.00129
AK006387	Me1 malic enzyme 1, NADP(+)-dependent, cytosolic	17436	−2.8	0.011484
AK005023	Sel1l sel-1 suppressor of lin-12-like (C. elegans)	20338	−2.25	0.003066
NM_001159375	Eif4a1 eukaryotic translation initiation factor 4A1	13681	−2.08	0.023416
AK002362	Myo5a myosin VA	17918	−2.24	0.00322
AK003786	Nfs1 nitrogen fixation gene 1 (S. cerevisiae)	18041	−2.05	0.009065
AK007618	Ak3 adenylate kinase 3	56248	−2.05	0.039247
NM_008303	Hspd1 heat shock protein 1 (chaperonin)	15528	−2.75	0.037992
NM_011631	Hsp90b1 heat shock protein 90, beta (Grp94), member 1	22027	−2.07	0.012751
NM_010516	Cyr61 cysteine rich protein 61	16007	−2.08	0.007693
NM_013614	Odc1 ornithine decarboxylase, structural 1	18263	−1.91	0.002118
NM_001111289	Caprin1 cell cycle associated protein 1	53872	−1.93	0.041035
NM_023908	Slco3a1 solute carrier organic anion transporter family, member 3a1	108116	−2.27	0.010399
NM_019699	Fads2 fatty acid desaturase 2	56473	−2.1	0.011032
AB046929	Chst7 carbohydrate (N-acetylglucosamino) sulfotransferase 7	60322	−2.15	0.000153
NM_033564	Mpv17l Mpv17 transgene, kidney disease mutant-like	93734	−2.04	0.004172
AK003671	Car3 carbonic anhydrase 3	12350	−2.4	0.020089
NM_032000	Trps1 trichorhinophalangeal syndrome I (human)	83925	−1.83	0.008962
AB031813	Slco1a1 solute carrier organic anion transporter family, member 1a1	28248	−5.5	0.024812
NM_019657	Hsd17b12 hydroxysteroid (17-beta) dehydrogenase 12	56348	−1.91	0.02581
NM_010302	Gna12 guanine nucleotide binding protein, alpha 12	14673	−1.82	0.010264
NM_010232	Fmo5 flavin containing monooxygenase 5	14263	−3.12	0.000843
AF319542	Kcnk5 potassium channel, subfamily K, member 5	16529	−2.11	0.038642
NM_001159555	Cd36 CD36 antigen	12491	−3.35	0.002187
NM_025903	Ifrd2 interferon-related developmental regulator 2	15983	−2.47	0.005388
AF133669	Arl6ip1 ADP-ribosylation factor-like 6 interacting protein 1	54208	−1.94	0.00508
BC022130	Slc26a1 solute carrier family 26 (sulfate transporter), member 1	231583	−1.85	0.008221
BC026422	Tgm1 transglutaminase 1, K polypeptide	21816	−2.49	0.000038
BC026598	Slc22a7 solute carrier family 22 (organic anion transporter), member 7	108114	−7.3	0.004741
M33324	Ghr growth hormone receptor	14600	−2.58	0.005396
M55333	Ace angiotensin I converting enzyme (peptidyl-dipeptidase A) 1	11421	−2.45	0.006298
NM_013876	Rnf11 ring finger protein 11	29864	−2.38	0.008352
NM_001122683	Bdh1 3-hydroxybutyrate dehydrogenase, type 1	71911	−1.88	0.001252
NM_009199	Slc1a1 solute carrier family 1 (neuronal/epithelial high affinity glutamate transporter, system Xag), member 1	20510	−2.07	0.004881

### Urinary Albumin Excretion Rate

The urinary albumin excretion rate was measured with a murine microalbuminuria ELISA kit (Exocell Inc., PA) according to the manufacturer’s suggestions.

**Figure 7:**
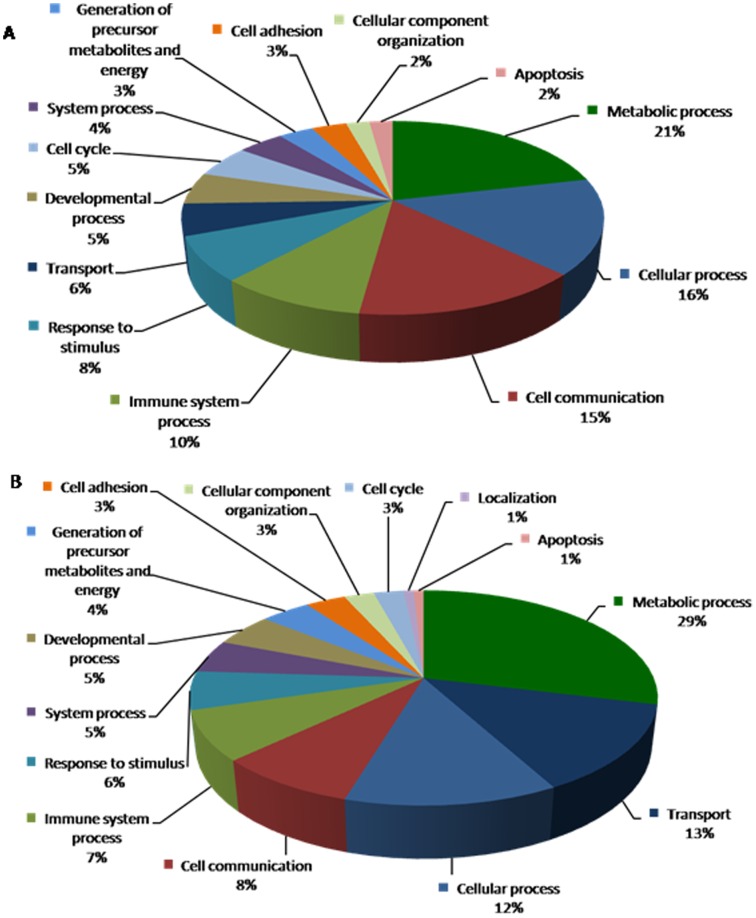
Biological processes depicting genes that are altered in response to diabetes in wild type control mice and in B_2_R^−/−^ null mice are shown in pie chart. Data compares genes altered in B_2_R^−/−^D vs. B_2_R^+/+^D (**A**) upregulated genes and (**B**) downregulated genes.

**Table 4:** Upregulated and Downregulated Genes in B_2_R^−/−^D vs. B_2_R^+/+^D.

Accession ID	Gene	Gene ID	Fold Change	P value
BC009155	Mgst1 microsomal glutathione S-transferase 1	56615	2.01	0.025734
NM_019423	Elovl2 elongation of very long chain fatty acids (FEN1/Elo2, SUR4/Elo3, yeast)-like 2	54326	2.08	0.003068
NM_021450	Trpm7 transient receptor potential cation channel, subfamily M, member 7	58800	1.83	0.002536
BC027434	Hbb-b2 hemoglobin, beta adult minor chain	15130	2.41	0.026002
NM_008218	Hba-a1 hemoglobin alpha, adult chain 1	15122	2.73	0.027316
AF047838	Clca1 chloride channel calcium activated 1	12722	2.15	0.00938
D89669	Cyp24a1 cytochrome P450, family 24, subfamily a, polypeptide 1	13081	6.34	0.054554
NM_008341	Igfbp1 insulin-like growth factor binding protein 1	16006	3.65	0.030456
NM_027884	Tns1 tensin 1	21961	1.77	0.007886
AAD38411	March7 membrane-associated ring finger (C3HC4) 7	57438	1.82	0.005006
NM_008182	Gsta2 glutathione S-transferase, alpha 2 (Yc2)	14858	2.05	0.020506
NM_011313	S100a6 S100 calcium binding protein A6 (calcyclin)	20200	2.51	0.003671
NM_011169	Prlr prolactin receptor	19116	2.6	0.018234
NM_010145	Ephx1 epoxide hydrolase 1, microsomal	13849	2.32	0.015186
NM_010424	Hfe hemochromatosis	15216	1.85	0.005272
NM_001172121	Rbms3 RNA binding motif, single stranded interacting protein	207181	2.35	0.034332
NM_010279	Gfra1 glial cell line derived neurotrophic factor family receptor alpha 1	14585	1.81	0.020694
BC013343	Hpd 4-hydroxyphenylpyruvic acid dioxygenase	15445	1.97	0.007464
NM_008096	Gc group specific component	14473	3.77	0.008264
BC023060	Efemp1 epidermal growth factor-containing fibulin-like extracellular matrix protein 1	216616	2.13	0.001006
AK009020	Clic3 chloride intracellular channel 3	69454	2.05	0.005867
NM_145942	Hmgcs1 3-hydroxy-3-methylglutaryl-Coenzyme A synthase 1	208715	2.75	0.005719
NM_019989	Sh3bgrl SH3-binding domain glutamic acid-rich protein like	56726	2.04	0.037097
NM_010169	F2r coagulation factor II (thrombin) receptor	14062	2.31	0.015754
NM_007569	Btg1 B-cell translocation gene 1, anti-proliferative	12226	2.03	0.025775
NM_001004148	Slc13a5 solute carrier family 13 (sodium-dependent citrate transporter), member 5	237831	1.86	0.029255
NM_145569	Mat2a methionine adenosyltransferase II, alpha	232087	1.8	0.034233
NM_001110831	Dnpep aspartyl aminopeptidase	13437	1.9	0.001443
NM_009837	Cct4 chaperonin containing Tcp1, subunit 4 (delta)	12464	2.06	0.000479
NM_009242	Sparc secreted acidic cysteine rich glycoprotein	20692	1.8	0.020269
NM_008597	Mgp matrix Gla protein	17313	1.91	0.002312
NM_019975	Hacl1 2-hydroxyacyl-CoA lyase 1	56794	2.17	0.003102
NM_054102	Ivns1abp influenza virus NS1A binding protein	117198	2.1	0.01779
NM_013806	Abcc2 ATP-binding cassette, sub-family C (CFTR/MRP), member 2	12780	2.18	0.019342
NM_031166	Id4 inhibitor of DNA binding 4	15904	2.36	0.013276
BC012874	Serpina1b serine (or cysteine) preptidase inhibitor, clade A, member 1B	20701	1.89	0.044292
NM_029023	Scpep1 serine carboxypeptidase 1	74617	1.84	0.001437
NM_009009	Rad21 RAD21 homolog (S. pombe)	19357	2.09	0.001454
NM_010362	Gsto1 glutathione S-transferase omega 1	14873	2.43	0.008124
NM_016792	Txnl1 thioredoxin-like 1	53382	1.84	0.016406
NM_011701	Vim vimentin	22352	1.95	0.026022
NM_007620	Cbr1 carbonyl reductase 1	12408	2.2	0.012532
AF108501	Clca2 chloride channel calcium activated 2	80797	2.57	0.003658
AF145253	Sec61a1 Sec61 alpha 1 subunit (S. cerevisiae)	53421	−2.11	0.005453
NM_013560	Hspb1 heat shock protein 1	15507	−2.36	0.004073
BC021352	Plod2 procollagen lysine, 2-oxoglutarate 5-dioxygenase 2	26432	−2	0.005958
NM_029550	Keg1 kidney expressed gene 1 [Mus musculus ]	64697	−1.8	0.031526
AK146840	Amd1 S-adenosylmethionine decarboxylase 1	11702	−2	0.014904
NM_030706	Trim2 tripartite motif-containing 2	80890	−2	0.048874
NM_010274	Gpd2 glycerol phosphate dehydrogenase 2, mitochondrial	14571	−1.9	0.008751
NM_008878	Serpinf2 serine (or cysteine) peptidase inhibitor, clade F, member 2	18816	−1.8	0.027152
NM_011435	Sod3 superoxide dismutase 3, extracellular	20657	−2	0.00248
BC006716	Vdr vitamin D receptor	22337	−1.8	0.005698
NM_019444	Ramp2 receptor (calcitonin) activity modifying protein 2	54409	−3.2	0.027003
AF067806	Pde8a phosphodiesterase 8A	18584	−1.9	0.036591
AF012834	Kcnj1 potassium inwardly-rectifying channel, subfamily J, member 1	56379	−2.1	0.025618
NM_007788	Csnk2a1 casein kinase 2, alpha 1 polypeptide	12995	−2	0.033254
L27424	Timp3 tissue inhibitor of metalloproteinase 3	21859	−2.1	0.0122
NM_008079	Galc galactosylceramidase	14420	−1.9	0.045887
NM_023646	Dnaja3 DnaJ (Hsp40) homolog, subfamily A, member 3	83945	−1.9	0.007017
NM_030721	Acox3 acyl-Coenzyme A oxidase 3, pristanoyl	80911	−2	0.018101
NM_018760	Slc4a4 solute carrier family 4 (anion exchanger), member 4	54403	−2.1	0.029658
NM_001164733	Mpp6 membrane protein, palmitoylated 6 (MAGUK p55 subfamily member 6)	56524	−2.1	0.023307
NM_010890	Mus musculus neural precursor cell expressed, developmentally down-regulated 4 (Nedd4)	17999	−1.9	0.000172
NM_010517	Igfbp4 insulin-like growth factor binding protein 4	16010	−2.2	0.018949
NM_008397	Itga6 integrin alpha 6	16403	−2.3	0.03713
NM_009203	Slc22a12 solute carrier family 22 (organic anion/cation transporter), member 12	20521	−1.9	0.010432
NM_018881	Fmo2 flavin containing monooxygenase 2	55990	−1.9	0.000286
NM_011851	Nt5e 5′ nucleotidase, ecto	23959	−2	0.020878
NM_001160404	Galnt1 UDP-N-acetyl-alpha-D-galactosamine:polypeptide N-acetylgalactosaminyltransferase 1	14423	−2	0.046958
NM_009443	Tgoln1 trans-golgi network protein	22134	−2.2	0.006538
BC019374	Gclc glutamate-cysteine ligase, catalytic subunit	14629	−1.8	0.014479
BC025936	Cyp4a12a cytochrome P450, family 4, subfamily a, polypeptide 12a	277753	−2.2	0.050246
BC021452	Ddx6 DEAD (Asp-Glu-Ala-Asp) box polypeptide 6	13209	−1.8	0.002793
AY038079	Fbxw11 F-box and WD-40 domain protein 11	103583	−2.3	0.013332
NM_016870	Acsm3 acyl-CoA synthetase medium-chain family member 3	20216	−2.7	0.073051
AF213670	Mlx MAX-like protein X	21428	−1.8	0.025547
NM_001164099	Add3 adducin 3 (gamma)	27360	−1.8	0.003839
NM_001167745	Wasl Wiskott-Aldrich syndrome-like (human)	73178	−2.3	0.005224
BC027063	Bdh1 3-hydroxybutyrate dehydrogenase, type 1	71911	−2.7	0.0021
AK003232	Cbr3 carbonyl reductase 3	109857	−2.57	0.049803
AK014338	Manf mesencephalic astrocyte-derived neurotrophic factor	74840	−1.9	0.027373
NM_001159375	Eif4a1 eukaryotic translation initiation factor 4A1	13681	−2.2	0.006428
NM_010911	Nfs1 nitrogen fixation gene 1 (S. cerevisiae)	18041	−2.1	0.002002
AK015410	Dnm2 dynamin 2	13430	−2.1	0.004377
AK013376	Aplp2 amyloid beta (A4) precursor-like protein 2	11804	−2.3	0.007349
AK007618	Ak3 adenylate kinase 3	56248	−2.2	0.026914
NM_031843	Dpp7 dipeptidylpeptidase 7	83768	−1.8	0.017748
EDL19081	Actb actin, beta	11461	−1.88	0.006181
NM_010477	Hspd1 heat shock protein 1 (chaperonin)	15510	−2.72	0.029635
NM_011631	Hsp90b1 heat shock protein 90, beta (Grp94), member 1	22027	−2.17	0.009681
NM_001111289	Caprin1 cell cycle associated protein 1	53872	−1.88	0.005003
NM_080555	Ppap2b phosphatidic acid phosphatase type 2B	67916	−2.08	0.001515
NM_011390	Slc12a7 solute carrier family 12, member 7	20499	−1.88	0.010937
NM_010302	Gna12 guanine nucleotide binding protein, alpha 12	14673	−1.83	0.001605
NM_019664	Kcnj15 potassium inwardly-rectifying channel, subfamily J, member 15	16516	−2.17	0.012327
U41465	Bcl6 B-cell leukemia/lymphoma 6	12053	−1.82	0.009357
AF319542	Kcnk5 potassium channel, subfamily K, member 5	16529	−2.05	0.003671
NM_008261	Hnf4a hepatic nuclear factor 4, alpha	15378	−1.8	0.00581
NM_001159555	Cd36 CD36 antigen	12491	−2.58	0.021768
NM_016697	Gpc3 glypican 3	14734	−2.43	0.001794
BB540964	Ifrd2 interferon-related developmental regulator 2	15983	−2.09	0.036417
BC022130	Slc26a1 solute carrier family 26 (sulfate transporter), member 1	231583	−1.81	0.011694
M33324	Ghr growth hormone receptor	14600	−2.71	0.020174
NM_013876	Rnf11 ring finger protein 11	29864	−2.37	0.002724
NM_026147	Rps20 ribosomal protein S20	67427	−1.97	0.023604
NM_008538	Marcks myristoylated alanine rich protein kinase C substrate	17118	−1.96	0.011844
NM_009199	Slc1a1 solute carrier family 1 (neuronal/epithelial high affinity glutamate transporter, system Xag), member 1	20510	−2.21	0.008478
U13836	Atp6v0a1 ATPase, H+ transporting, lysosomal V0 subunit A1	11975	−1.9	0.007939

### Systems Biology Analysis

The microarray differential expression of the wild type B_2_R vs. knockout (B_2_R^−/−^) in control and diabetic phenotypes was further analyzed using a systems biology approach to assess the altered pathway(s) relevant to differential B_2_R knockout (B_2_R^−/−^) phenotype mice and its contribution to the development of Diabetes. PathwayStudio software (v 9.0; Ariadne Genomics, Rockville, MD, USA) was applied for the systems biology analysis. This software helps to interpret biological meaning from differential gene expression, build and analyze pathways, and identify altered cellular processes and molecular functions involved. PathwayStudio comes with a built-in resource named ResNet, which is a database of molecular interactions based on natural language processing of scientific abstracts in PubMed.

**Figure 8:**
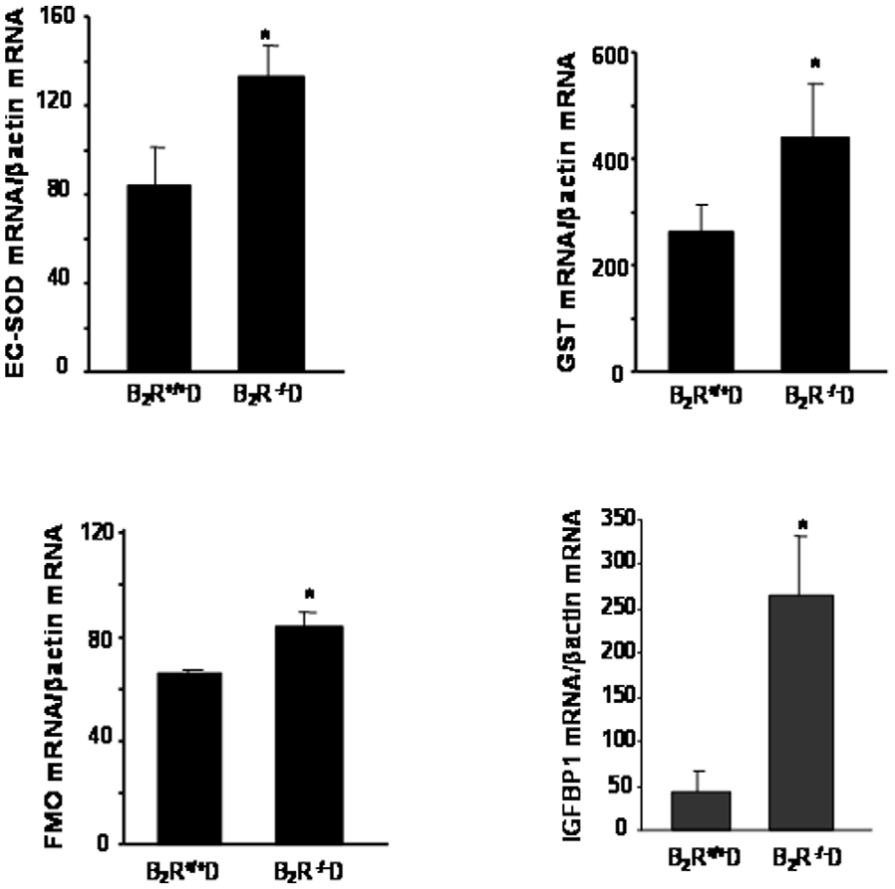
Renal expression of (A) Superoxide dismutase 3, extracellular (EC-SOD), (B) Glutathione S-transferase, (GST), (C) Flavin containing monooxygenase (FMO) and (D) Insulin-like growth factor binding protein (IGFBP-1) in B_2_R^+/+^D and B_2_R^−/−^D. Renal cortex mRNA levels were measured by real time PCR. Data presented in the bar graph demonstrates that disruption of B_2_R results in significant increases in anti-oxidant enzymes as well as IGFBP-1 (*P<0.05 B_2_R^−/−^D vs. B_2_R^+/+^D, n = 3).

For gene ontology analysis including differential molecular function and biological processes involved, PANTHER software (Protein ANalysis THrough Evolutionary Relationships; http://www.pantherdb.org/genes/batchIdSearch.jsp) was utilized to classify proteins into distinct categories of molecular functions and biological processes. Panther software uses published scientific experimental evidence and evolutionary relationships abstracted by curators with the goal of predicting function even in the absence of direct experimental evidence. Proteins are classified into families and subfamilies of shared function, which are then categorized using a highly controlled vocabulary (ontology terms) by biological process, molecular function and molecular pathway.

**Figure 9:**
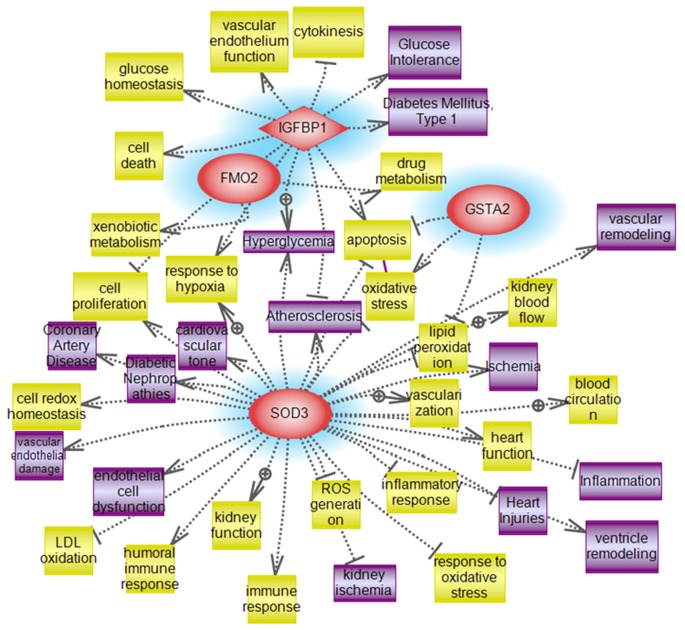
Pathways influenced by the validated targeted genes. Targeted system biology analysis of the biological process and molecular function of the 4 validated genes (Superoxide dismutase 3, extracellular (EC-SOD), Glutathione S-transferase, alpha 2(Yc2) (GST-Yc2), Flavin containing monooxygenase 2 (FMO2), Insulin-like growth factor binding protein (IGFBP-1). Similar to the identified altered pathways, these 4 proteins are shown to be related to the identified molecular pathways (apoptosis, oxidative stress and inflammation).

## Statistical Methods

### Power Analysis

Sample size calculation for our study was determined by using the formula by Hedeker D et al, for longitudinal data [14]. In this study we assumed 80% power, significance of 5%, repeated measure correlation of 0.5, 9 measurement time points, within subject variance of 4.2, and medium effect size of 0.3. This resulted in 2.3 mice per group, and accounting for possible attrition effect we inflated our sample size by 20% so the sample size in each group will be 2.76 mice.

**Figure 10:**
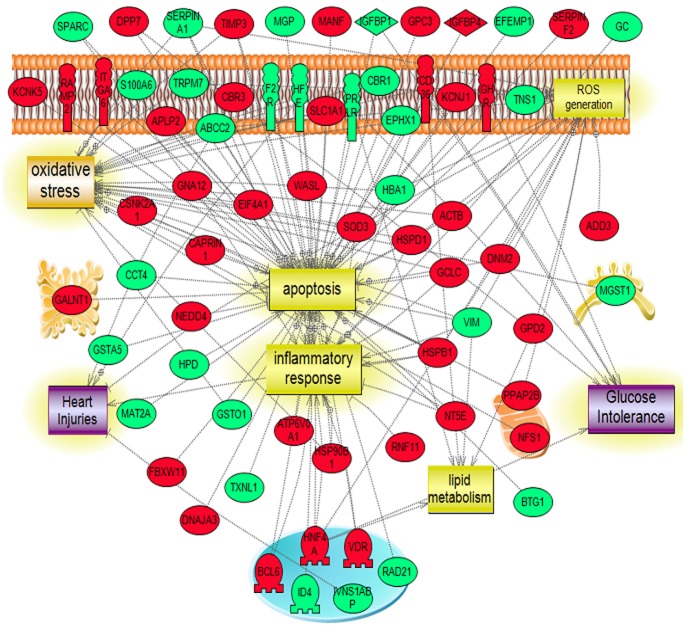
Molecular & Biological Pathway Interaction Map Analysis upon Diabetes induction with or without disruption of B_2_R. Using Pathway Studio 9.0, altered genes relevant to diabetic induction with or without disruption of B_2_R. were analyzed. In B_2_R^−/−^D vs. B_2_R^+/+^D mice, a total of 109 genes were found to be altered (43 upregulated and 66 downregulated). The network was generated using “direct interaction” algorithm to map cellular processes and interactions among altered genes. Of interest, global Pathway analysis revealed association of these genes to oxidative stress mechanisms (ROS generation & oxidative stress), cardiac injury mechanisms along with pronounced inflammatory process with a marked alteration in the pro-apoptotic genes. The upregulated genes are shown in green and downregulated genes are in red.

### Statistical Analysis

Results are expressed as mean ± standard error, unless stated otherwise. All data were analyzed using SAS (SAS Institute Inc., Version 8, Cary, NC). t-tests were used to analyze continuous outcomes versus each covariate separately. To compare means values across three or more groups, ANOVA was used. Generalized linear models and generalized estimating equations were used to compare albumin excretion rates, plasma glucose levels and body weights within mice and across groups over time. A longitudinal data analysis was conducted to assess the effect of group on the AER levels over time. A mixed model was fit and spatial data covariance structure was used to accommodate for the unequally- spaced measurement time points. In this context, a continuous-time model was employed using variance-covariance matrix with type = sp (pow) in SAS PROC MIXED. Bonferroni correction was used to adjust for inflated type I error when making multiple comparisons. Statistical significance was determined using a two-sided test and significance was assumed for P-values ≤0.05.

## Results

### Characteristics of the Diabetic State

Plasma glucose levels were markedly elevated 2 weeks after STZ injection in both B_2_R^+/+^D and B_2_R^−/−^D groups of mice compared to their non-diabetic controls, and remained elevated throughout the study period (Figure 1A). On average plasma glucose levels increased by 205 mg/dl in B_2_R^−/−^D null mice and by 251 mg/dl in B_2_R^+/+^D null mice compared to B_2_R^+/+^C mice, P<0.001. No significant difference in plasma glucose levels was observed between B_2_R^+/+^C mice and B_2_R^−/−^C mice, P = 0.276. No significant time effect on plasma glucose level was observed, P = 0.2647. Also no significant effect of group by time interaction on plasma glucose levels was detected, P = 0.28. Hence, the observed difference in plasma glucose levels across groups was primarily due to group effect.

Initial body weights were not significantly different between diabetic and non-diabetic mice. However, B_2_R^−/−^D mice had significantly reduced bodyweight after 14 weeks and B_2_R^+/+^D after 20 weeks compared with B_2_R^+/+^ C and B_2_R^−/−^ C mice and this reduction in body weight was maintained for the duration of the study (Figure 1B). Body weight analyses revealed that there was no significant group effect on bodyweights over time, but there was a significant effect of time on bodyweights, P = 0.0011. In addition, there was interaction between time and group effect on changes in body weights P = 0.0011. Thus, the decrease in bodyweights in B_2_R^−/−^D null mice and B_2_R^+/+^D mice compared to B_2_R^+/+^C mice are a result of time effect.

### Albumin Excretion Rate

The albumin excretion rate results are presented in Figure 2. Groups were defined as B_2_R^+/+^C, B_2_R^+/+^D, B_2_R^−/−^C and B_2_R^−/−^D. AER was modeled with a time and group main effect and a time by group effect. Since AER in each mouse was measured up to 10 times over 23 weeks, a longitudinal data analysis was conducted to assess the effect of group on the AER levels over time. A mixed model was fit and spatial data covariance structure was used to accommodate for the unequally-spaced measurement time points. Our results showed that there was a significant overall group effect with P<0.0001. In particular, when the wild type control group B_2_R^+/+^C was considered as the reference group, we observed that B_2_R^−/−^D had a significant increase in the AER by 13.5 mg/24 h, P = 0.001. Overall, a significant increase by about 28.5 mg/24 h in AER was also observed for B_2_R^+/+^D mice compared to B_2_R^+/+^C mice P<.0001. No significant differences in AER was observed between B2R^+/+^C and B2R^−/−^C, P = 0.1629.

Our result also showed that the B_2_R^−/−^D null mice had a significant decrease of 14.97 mg/24 h in the AER levels compared to wild type B_2_R^+/+^D mice, P = 0.0005. Some minor time effect on the AER was also observed. In particular, we can estimate that overall the AER appeared to be decreasing with time at a slow rate of 0.547 mg/24 h, P<.0001. An interaction test was then performed which showed that there is no significant interaction between time and group (P-value  = 0.24). Although there was some minor effect of time on AER, the observed changes in AER across groups was mainly due to group effect rather than an effect of time.

### Hierarchical Clustering of Gene Expression

Differential gene expression profiles in the kidney were identified among four groups of mice: B_2_R^+/+^C, B_2_R^+/+^D, B_2_R^−/−^C and B_2_R^−/−^D. Each column represents one sample, and the color bars represent the median value of three array experiments for an individual mouse for that gene (Figure 3).

### Gene Regulation in Response to Disruption of B_2_R

Upon deletion of B_2_R, There were a total of 14 altered genes (4 upregulated and 9 downregulated shown in **Table 1**); these include genes that code for ATPase activity, hemoglobin and enzymes involved in protein metabolism. Among the altered genes, Monoglyceride lipase (MGLL; EC 3.1.1.23) and lysine (K)-specific demethylase 2B (KDM2B) were found to be downregulated due to B_2_R deletion. KDM2B gene encodes a member of the F-box protein family lysine (K)-specific demethylase 2B which function in phosphorylation-dependent ubiquitination while MGLL gene functions together with hormone-sensitive lipase to hydrolyze intracellular triglyceride stores in adipocytes and other cells to fatty acids and glycerol. The biological processes depicting genes that are altered in response to B_2_R disruption are shown in **Figures 4** **A** and **B.**

### Gene Regulation in Response to Diabetes

Upon Diabetes induction, a total of 9 genes were found to be upregulated and 16 genes downregulated compared to B_2_R^+/+^C wild type mice. An enriched pathways analysis identified genes associated with potassium transport, cell cycles and lipid metabolism as shown in **Table 2**). The biological processes depicting genes that are altered in response to diabetes are shown in **Figures 5A and B** **.**

Of great interest, in B_2_R^−/−^ null mice, a total of 181 genes were regulated by diabetes including 91 upregulated genes and 90 downregulated genes, respectively (**Table 3**). A thorough systems biology analysis of specific enriched pathways, several genes were found to be associated with: endothelial cellular injury, insulin & lipid metabolism, oxidative stress, cardiac and kidney toxicity as illustrated in the biological processes (**Figures 6A & B**).

### Gene Expression in Diabetes with or without Disruption of B_2_R

In B_2_R^−/−^D vs. B_2_R^+/+^D mice, a total of 43 genes were upregulated and 66 genes were downregulated (**Table 4**). Among these altered genes: IGFBP, GST, EC-SOD and GHR genes. In a detailed assessment of these genes, gene expressions of IGFBP-1(3.65 fold) and GST (Yc2, 2.05 fold; omega1, 2.43 fold) were elevated in the B_2_KR^−/−^D mice compared to the B_2_KR^+/+^D mice. On the other hand, gene expressions of Insulin-like growth factor-binding protein-4 (IGFBP-4) (−2.18 fold), EC-SOD (−1.95 fold), FMO2 (−1.94 Fold) and GHR (−2.7 fold) were suppressed in the B_2_KR^−/−^D mice compared to the B_2_KR^+/+^D mice, P<0.05. The biological processes depicting genes that are altered in response to diabetes +/− B_2_R are shown in Figure 7A & B.

### Validation of Specific Gene Expressions by Quantitative Real-time PCR Superoxide Dismutase 3, Extracellular (EC-SOD)

EC-SOD gene encodes a member of the superoxide dismutase (SOD) protein family which are antioxidant enzymes that catalyze the dismutation of two superoxide radicals into hydrogen peroxide and oxygen protecting from oxidative stress. EC-SOD expression tended to be suppressed by diabetes in the wild type mice. Interestingly, in the B_2_R^−/−^D mice, EC-SOD expression was increased up to 37% compared to that in the B_2_R^+/+^D mice (*P<0.05 vs. B_2_R^+/+^D, Figure **8A**).

### Glutathione S-transferase, Alpha 2(Yc2) (GST-Yc2)

GST-Yc2 catalyze the conjugation of reduced glutathiones and a variety of electrophiles, including many known carcinogens and mutagens. Our data indicated that the expression of GST was significantly higher in B_2_R^−/−^D mice compared to B_2_R^+/+^D mice (*P<0.05 vs. B_2_R^+/+^D, Figure 8B).

### Flavin Containing Monooxygenase 2 (FMO2)

FMO2 family is NADPH-dependent enzymes that catalyze the oxidation of many drugs and xenobiotics. In the B_2_R^+/+^D mice, FMO2 expression was decreased up to 34% compared to that in the controls. However, the expression FMO2 was significantly higher in B_2_R^−/−^D mice compared with B_2_R^+/+^D mice (*P<0.05 vs. B_2_R^+/+^D, Figure 8C).

### Insulin-like Growth Factor Binding Protein (IGFBP-1)

IGFBP-1 gene is a member of the insulin-like growth factor binding protein (IGFBP) family and encoding proteins with an IGFBP domain and a thyroglobulin type-I domain. It binds both insulin-like growth factors (IGFs) I and II and circulates in the plasma prolonging the half-life of the IGFs. In our work, the deletion of B_2_R didn’t change the expression of IGFBP-1. However, IGFBP-1 expression was decreased up to 33% by diabetes in the wild type mice (P<0.05). Interestingly, in B_2_R^−/−^D mice, IGFBP-1 expression was upregulated significantly: up to 2.7-fold increase compared to that in B_2_R^+/+^D (*P<0.05 vs. B_2_R^+/+^D, Figure 8D).

We next performed a targeted analysis to identify the involvement of these selected validated genes in the most highlighted altered pathways (apoptosis, oxidative stress and inflammation). These genes were shown to be highly related to the aforementioned pathways as shown in **Figure 9**.

### Systems Biology Analysis of Altered Genes in B_2_R^−/−^D and B_2_R^+/+^D mice

Pathway Studio 9.0 (2011, Ariadne Genomics, Rockville, MD) was also used to search for potential altered cellular processes, and related pathways for associations with gene alterations in our diabetic mice in the presence or absence of B_2_R. The network was generated using the “direct interaction” algorithm with the filters of “*Cellular process and Protein*” as Entity Type while the Relation Type parameter was set to “*Regulation Analysis*” to map altered pathways regulated by the identified (downregulation vs. upregulation) subsets of genes. Several processes believed to be central to the pathogenesis of DN included oxidative stress mechanisms (ROS generation & oxidative stress), cardiac injury mechanisms along with pronounced inflammatory process with a marked alteration in the pro-apoptotic genes as illustrated in **Figure 10** **.**

## Discussion

A pivotal event initiated by DN is glomerular injury, characterized by mesangial deposition and podocyte loss. The degree of podocyte loss and mesangial expansion are strongly correlated with the clinical manifestations of DN, such as albuminuria and decreased GFR [3], [4], [15]. Microalbuminuria, an early marker of DN, signifies high risk for progressive renal failure and cardiovascular disease [16]. Microalbuminuria has also been associated with increased cardiovascular mortality in diabetic and non-diabetic populations and with generalized and glomerular endothelial dysfunction [17]. Identifying biomarkers and risk factors that contribute to the development of microalbuminuria may provide insights into the mechanisms of diabetic renal injury.

Few interventions have been shown to slow the progression of renal disease in diabetic patients. These include intensive glycemic control, blood pressure control and treatment with angiotensin converting enzyme inhibitors (ACEI) or angiotensin receptor blockers (ARBs) [6], [18]. Despite these interventions and beneficial effects, diabetic patients progress with time to develop end stage renal disease. It is of significance to note here that a recent Interventional study aimed at blockade of the renin-angiotensin system (RAS) with ACE-inhibitors or ARBs, in patients with type 1 diabetes, did not slow nephropathy progression [19]. However, the exact factors responsible for these maladaptive signals leading to renal failure are poorly defined.

Metabolic imbalances associated with high tissue glucose and abnormal lipid levels in the diabetic state influence many pathways that contribute to the pathogenesis of DN [20], [21]. The modifiable factors engaged in these processes are yet to be identified but there is evidence for promotion of chronic low-grade inflammation, oxidative stress, endothelial dysfunction, stimulation of proliferative/apoptotic pathways, and deposition of extracellular matrix [22]–[24]. Importantly, inflammatory mediators and growth factors are increasingly recognized as key players in the pathogenesis of DN [25]–[27].

Our published work has provided evidence for the involvement of the kallikrein-kinin system (KKS) in the initiation of DN [7], [13]. In the current work, we performed longitudinal data analysis to assess the rate of change in AER levels over time among the 4 different groups. Our data indicated that targeted deletion of B_2_R in mice interferes with the progression of DN. Diabetic B_2_R^−/−^ mice display reduced AER compared to diabetic B_2_R^+/+^ mice. Other investigators have also implicated a role for B_2_R in DN. Polymorphisms in the human B_2_R have been linked to increased albuminuria in diabetic patients and to the development of chronic renal failure [28], [29]. In addition, blockade of B_2_R markedly reduced the proteinuria in STZ-diabetic mice and inhibition of B_2_R ameliorated the accelerated nephropathy in uninephrectomized db/db mice, lending support to the pathogenic role of B_2_R in DN [30], [31].

Contrary to the aforesaid findings, Kakoki and Smithies have reported a protective role for B_2_R in DN. They have shown that the insulin Akita (Ins2^Akita^) mice crossed with null B_2_R (In2^Akita^/B_2_R^−/−^) or with double-null B_2_R and B_1_R (In2^Akita^/B_2_R^−/−/^B_1_R^−/−^) displayed increased albuminuria compared to Ins2^Akita^ mice alone [32], [33].Other factors contributing to these apparent differences in the role of B_2_R in DN may be attributed to differences in the model of DN studied, genetic background of the animal models studied, severity and metabolic control of the diabetic state, specifics of the experimental design, the end points measured. It is noteworthy to point here that a confounding factor to be considered when using the Insulin Akita mouse is the propensity for these mice to develop mesangial deposits of IgG [34].

To investigate the underlying mechanisms and involved pathways linking the role of B_2_R genotype to the development/progression of DN, we examined the contribution of B_2_R genotype on the global genomics level. We performed a global microarray study comparing gene expression profiles among four groups of mice respectively: (B_2_R^+/+^C, B_2_R^+/+^D, B_2_R^−/−^C and B_2_R^−/−^D). Findings from this work highlighted the role of several altered pathological pathways involved in the development of diabetes in the B_2_R^−/−^D vs. B_2_R^−/−^C mice which included: endothelial injury, oxidative stress, and insulin and lipid metabolism.

A detailed analysis of the top scoring biological processes data [*Panther Analysi*s] reflected the central role of B_2_R to increased immune response/inflammation along with other cellular functions (transport, systems process and response to stimulus which can be linked to protective/compensatory mechanism. This is in accordance with a previous study by Bascands et al, in which a global microarray renal gene expression changes were examined in lipopolysacharide-treated wild-type and kinin B_1_ receptor-knockout mice to investigate underlying mechanisms of renal inflammation reflected the role of acute phase response and inflammatory process [35].

This is in contrast to the sole effect of diabetes induction in wild type mice which reflected more pronounced metabolic/cellular processes changes (metabolites precursor generation, cellular adhesion, and cellular communication) rather than inflammatory immune response mediated response. Of interest, is the upregulation of one of the genes, aquaporin 4, (AQP4, 2.24) due to diabetes. AQP4 functions as a water transport channel in the kidney and has been shown to be downregulated in mice lacking B_2_R [36].

These results validate existing published literature linking renal inflammation to early events of renal disease [37]–[39]. Furthermore, a global systems biology analysis among the diabetic mice with or without disruption of B_2_R **(**B_2_R^−/−^D vs. B_2_R^+/+^D) illustrated the role of oxidative stress mechanisms (ROS generation & oxidative stress), along with inflammatory process with a marked alteration in the pro-apoptotic genes. Indeed, these results may reflect a pathologic exacerbative role of B_2_R in inducing cellular vascular injury mediated via apoptotic pathways in the presence of diabetes. These findings are in concert with other microarray studies involving B_1_ and B_2_ receptor knockout mice [40], [41].

Taken together, the finding of this study investigates the contributing role of B_2_-receptors in either exacerbating or at least enhancing the occurrence of diabetic nephropathy. In conclusion, the present study investigates the impact B_2_R deletion on the development of DN. A critical analysis of the data hints that renal function is preserved in the B_2_R^−/−^D mice especially at the early stages of DN, compared to that of B_2_R^+/+^D mice; these data were substantiated by the genomics/systems biology analysis. To the best of knowledge, this represents the first study that utilizes wide scale genomic/systems biology analysis in B_2_R^−/−^D mice. Finally, several of the identified genes (EC-sod, GST, IGFBP1 and FMO) were validated with RT-PCR to confirm gene alteration. Further studies including immunohistological analysis and assessment of protein levels and the activities of the antioxidants identified are certainly necessary to further evaluate the contributing role of the disruption of the B_2_-receptors.
